# Weil's Syndrome Masquerading as Acute Cholangitis: A Case Report of a Farmer With Leptospirosis

**DOI:** 10.1002/ccr3.73751

**Published:** 2026-10-16

**Authors:** Ahmed Ali Shaikh, Fiza Shaikh, Saad Arshad, Tarachand Devrajani, Humayou Ahmed, Manal Al‐Dhaheri

**Affiliations:** ^1^ Liaquat University of Medical and Health Sciences Jamshoro Pakistan; ^2^ Peoples University of Medical and Health Sciences Nawabshah Pakistan; ^3^ Dow University of Health Sciences Karachi Pakistan; ^4^ Sana'a University, Faculty of Medicine and Health Sciences Sana'a Yemen

## Abstract

Leptospirosis can masquerade as acute cholangitis with cholestatic jaundice, fever, and leukocytosis. In febrile patients with cholestatic liver profile without biliary obstruction, especially farmers with calf pain and conjunctival suffusion, consider leptospirosis early to initiate timely antibiotics and prevent poor outcomes.

## 1 Introduction

Leptospirosis is a globally distributed bacterial zoonosis caused by pathogenic species of the genus Leptospira, primarily affecting populations in areas of socioeconomic vulnerability [1]. It commonly infects animal hosts, such as rodents, which shed the pathogen in their urine. Humans become infected through occupational or recreational exposure with contaminated water or soil, with entry occurring via abraded skin or mucous membrane [2]. Leptospirosis presents significant diagnostic challenges, particularly in its early stages, due to several factors such as nonspecific clinical presentation, including fever, muscle pain, and headache, which resemble those of other acute febrile illnesses (AFIs) prevalent in endemic regions, such as dengue, influenza, and malaria [3]. Approximately 10% of patients with leptospirosis develop severe disease. The pathogenesis is believed to involve a vasculitic process, in which both direct tissue injury caused by Leptospira and immune‐mediated mechanisms contribute to endothelial dysfunction, deranged microcirculation, and multi‐organ damage. Severe leptospirosis may present as Weil's syndrome, classically characterized by conjunctival suffusion, jaundice, and acute kidney injury. Other manifestations include pulmonary involvement, hepatic dysfunction, renal impairment, cardiac complications, hematological abnormalities, and gastrointestinal involvement such as pancreatitis and acalculous cholecystitis [4]. We report a case of leptospirosis presenting with clinical features of acute cholangitis in line with CARE guidelines [5], highlighting the need to consider leptospirosis in the differential diagnosis of cholestatic jaundice when biliary obstruction is absent, particularly in endemic regions and occupationally exposed individuals.

## 2 Case History and Examination

A 35‐year‐old male farmer with a 10‐pack‐year smoking history and alcohol intake of 1–2 glasses per day for 5–7 years; however, he could not recall the exact type of alcoholic beverage, serving size, or alcohol concentration (abstinent for the past 5 months) presented with a 7‐day history of fever, abdominal pain, and bilateral lower limb pain. The abdominal pain was localized to the upper abdomen and was not associated with abdominal tenderness. He also reported the yellow‐red discoloration of the sclera, dark urine, pale stools, and generalized body itching. There was no history of biliary colic, known gallstones, or hepatotoxic medication use.

Based on these findings, the patient was admitted to the medical ward for detailed evaluation and further investigations. On arrival, the patient was hypotensive with a blood pressure of 85/50 mmHg, with a pulse rate of 90 beats/min, respiratory rate of 28 breaths/min, oxygen saturation of 95% on room air, and temperature of 98.8°F. On physical examination, the patient had marked icterus with conjunctival suffusion (Figure 1) without any peripheral stigmata of chronic liver disease. The abdomen was soft and nontender, with no right upper quadrant tenderness, guarding, or rigidity. There was no organomegaly, no lymphadenopathy and the remainder of the systemic examination was unremarkable. Although the patient had fever and jaundice with upper abdominal pain, he did not fulfill Charcot's triad because abdominal tenderness was absent.

**FIGURE 1:**
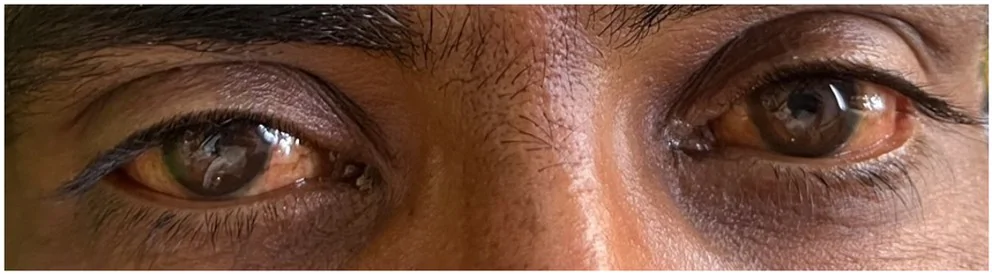
Marked icterus with conjunctival suffusion in a 35‐year‐old farmer with Weil's syndrome.

## 3 Differential Diagnosis, Investigations and Treatment

Initial laboratory investigations (Table 1) showed conjugated hyperbilirubinemia (total bilirubin 10.6 mg/dL, direct fraction 9.81 mg/dL) with a cholestatic pattern (ALP 267 U/L, GGT 105 U/L) and only mild ALT elevation (58.4 U/L). Furthermore, TLC count was 13.8 × 10^3^/μL, neutrophil count 92.4%, platelet count of 20 × 10^3^/μL, along with ESR of 95 mm/h and CRP of 223 mg/L suggesting significant systemic inflammatory response supportive of acute cholangitis. However, blood urea was 169 mg/dL and creatinine 4.97 mg/dL indicated significant renal impairment. The patient also had oliguria in the setting of acute kidney injury. Viral serologies (HBsAg, anti‐HCV, HIV), dengue NS1 antigen, and malaria tests (ICT and smear) were negative. Blood culture was also negative. Apart from blood culture, which was negative, no additional tests for typhoid fever such as Typhidot or Widal were carried out (Table 1). Abdominal ultrasonography revealed no hepatobiliary abnormality, with a common bile duct diameter of 0.4 cm showing no evidence of biliary obstruction, rendering acute cholangitis substantially less likely. Chest X‐ray was unremarkable (Figure 2).

**TABLE 1:** Baseline laboratory investigations at presentation.

Parameter	Result at presentation	Reference range
Hematology		
Hemoglobin (g/dL)	13.8	13–18
MCV (fL)	83.5	76–96
Total leukocyte count (×10^3^/μL)	13.8	4–11
Neutrophils (%)	92.4	50–70
Lymphocytes (%)	4.1	25–45
Platelet count (×10^3^/μL)	20	150–400
ESR (mm/h)	95	0–25
CRP (mg/L)	223	< 5
Liver function tests		
Total bilirubin (mg/dL)	10.6	0.3–1.2 (adults)
Direct bilirubin (mg/dL)	9.81	< 0.3
ALT/SGPT (U/L)	58.4	< 50
Alkaline phosphatase (U/L)	267	30–120
GGT (U/L)	105	8–61
Renal function and electrolytes		
Blood urea (mg/dL)	169	20–40
Serum creatinine (mg/dL)	4.97	0.7–1.2
Serum electrolytes (Na^+^, K^+^, Cl^−^)	Within normal limits	—
Viral serology		
HBsAg	Non‐reactive	—
Anti‐HCV	Non‐reactive	—
HIV	Non‐reactive	—
Infectious disease screening		
Dengue NS1 antigen	Negative	—
Malaria ICT	Negative	—
Malarial parasite smear	Negative	—
Coagulation profile		
PT (seconds)	15.4	9.7–12.8
APTT (seconds)	46	22–32
INR	1.28	0.9–1.2
Urinalysis		
Urine protein	2+	Negative
Urine pH	5.5	5.0–8.0
Pus cells	Present	0–5/HPF
Blood	Negative	Negative
Bacteria	Mild bacteria present	None
Granular casts	Present	Absent
Hyaline casts	Negative	Absent
Epithelial cells	Negative	Few/Absent

**FIGURE 2:**
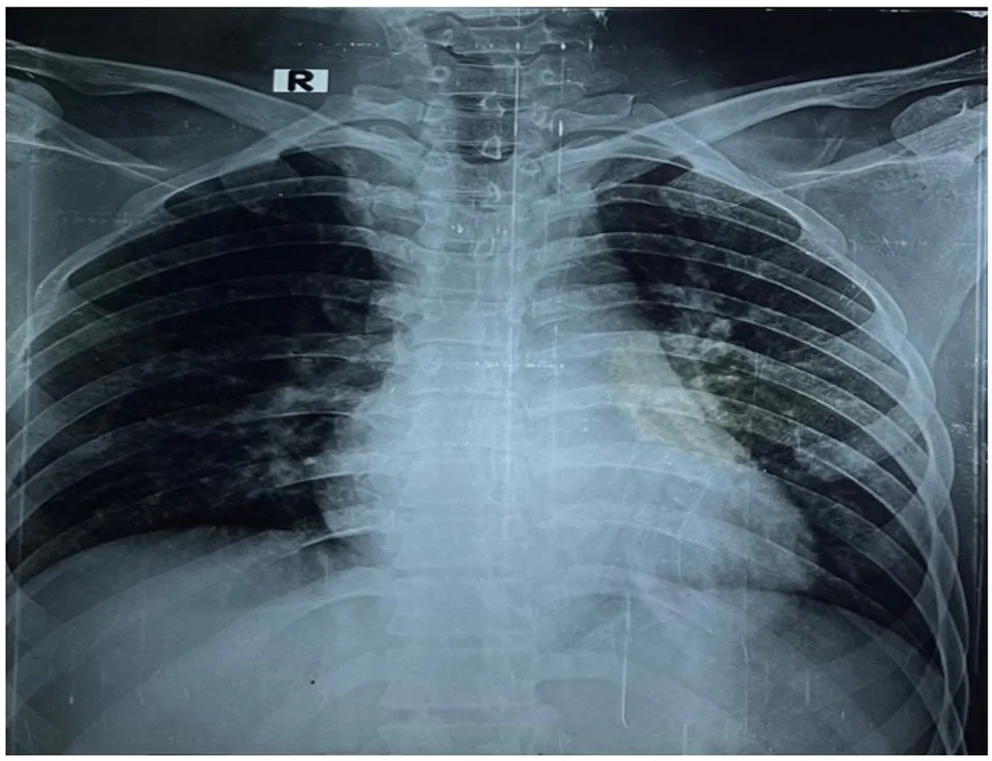
Chest radiograph showing clear lung fields with no consolidation or pleural effusion.

Differential diagnoses that were considered at the time of presentation included acute cholangitis, dengue, malaria, sepsis with multi‐organ failure, viral hepatitis, drug‐induced liver injury, and other tropical febrile illnesses. Viral hepatitis was excluded on the basis of negative HBsAg and anti‐HCV serology. Drug‐induced liver injury was considered unlikely in the absence of any past or current history of hepatotoxic medication use. Malaria and dengue were excluded in view of the negative laboratory results (Malaria ICT and smear, dengue NS1 antigen) and sepsis with multi‐organ failure was excluded on the basis of negative blood culture. In view of the combination of cholestatic jaundice, acute kidney injury, thrombocytopenia, and conjunctival suffusion, acute cholangitis became less likely and leptospirosis was considered a superior differential diagnosis. This diagnosis was further supported by the fact that the patient had bilateral leg pain, more specifically calf muscle pain, and that the patient is a farmer with repeated exposure to wet fields, which is strongly suggestive of leptospirosis.

To support the diagnosis, serum anti‐leptospiral IgM was tested via ELISA, which was positive with an index value of 2.18, consistent with acute leptospirosis (Table 2). Confirmatory testing by polymerase chain reaction (PCR) or microscopic agglutination testing (MAT) was unavailable at our center. Although IgM ELISA supports the diagnosis in the appropriate clinical context, it has recognized limitations, including possible cross‐reactivity; therefore the result was interpreted in conjunction with the characteristic clinical and laboratory features.

**TABLE 2:** Leptospirosis‐specific diagnostic workup.

Investigation	Method	Result	Reference range
Serum anti‐Leptospira IgM antibody	ELISA	2.18	Negative: < 0.9; Equivocal: 0.9–1.1; Positive: > 1.1
Serum Creatine phosphokinase (CPK)	Standard biochemical assay	39 U/L	39–308 U/L

Given the marked conjugated hyperbilirubinemia, elevated urea and creatinine and strong clinical suspicion of both leptospirosis and acute cholangitis, empirical treatment was initiated at the time of admission. The patient received IV ceftriaxone twice daily for 4 days and IV normal saline for adequate hydration. Intravenous ceftriaxone was chosen because it provides reliable coverage for common biliary pathogens while also constituting an established first‐line parenteral agent for severe leptospirosis. Other agents such as penicillin G or doxycycline are used to be effective in milder disease; however, third‐generation cephalosporins are preferably given in severe disease with multi‐organ involvement. IV Paracetamol was prescribed on an as‐needed basis for fever and pain; however, the patient only had a single episode of fever during the 4‐day hospital stay. Following discharge, he was advised to complete a further 3 days of ceftriaxone 1 g intravenously twice daily at a local peripheral daycare facility. In total, IV ceftriaxone 1 g twice daily for 4 days in hospital plus 3 days after discharge (total 7 days) was given.

## 4 Outcome and Follow‐Up

Serial laboratory investigations (Table 3) demonstrated progressive improvement in renal function, liver function, leukocyte count, and platelet count following initiation of therapy (Figure 3). As the patient remained hemodynamically stable and did not develop any bleeding despite severe thrombocytopenia, platelet, FFPs, and cryoprecipitate were not administered, and the patient was managed conservatively under close clinical monitoring. Follow up at 1 week after completion of antibiotics, the patient reported complete resolution of fever, jaundice, abdominal pain, and calf myalgia. Repeat laboratory testing showed near‐normalization of bilirubin, renal parameters, leukocyte count, and platelet count. Long‐term follow‐up beyond this point was not available.

**TABLE 3:** Serial laboratory trends during hospitalization.

Parameter	Day 0 (at the time of admission)	Day 1	Day 2	Day 3	Reference range
Total leukocyte count (×10^3^/μL)	13.8	15	13	10.9	4–11
Neutrophils (%)	92.4	90	79	73	50–70
Platelet count (×10^3^/μL)	20	22	31	55	150–400
Blood urea (mg/dL)	169	196	162	85	20–40
Serum creatinine (mg/dL)	4.97	4.5	3.6	2	0.7–1.2 (males)
Total bilirubin (mg/dL)	10.6	10.5	10.7	6	0.3–1.2 (adults)
ALT/SGPT (U/L)	58.4	56	41	47	< 50 (males)
Alkaline phosphatase (U/L)	267	250	202	202	30–120 (males)

**FIGURE 3:**
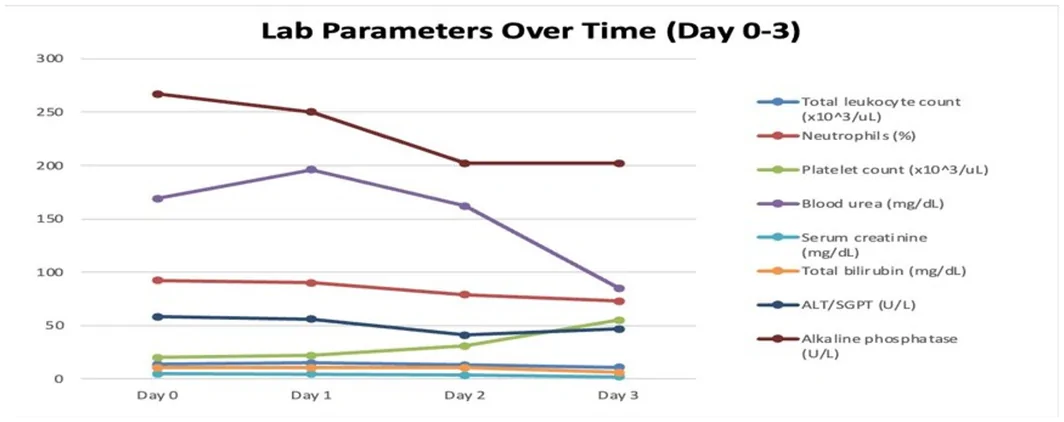
Serial laboratory trends showing progressive improvement in bilirubin, creatinine, leukocyte count, and platelets after initiation of ceftriaxone.

## 5 Discussion

This case illustrates a diagnostically challenging presentation of leptospirosis mimicking acute cholangitis, underscoring the need for broad differentials in patients presenting with cholestatic jaundice, particularly in endemic and occupationally exposed populations.

Leptospirosis is among the leading zoonotic causes of morbidity and mortality. Studies reveal significantly increased cases and disproportionate transmission rates in lower socioeconomic communities due to inadequate sanitation infrastructure and/or lack of enforcement of safe drinking water standards [6]. Leptospirosis diagnosis presents several challenges due to its variable clinical presentation, as many cases of leptospirosis are mild or asymptomatic, leading to them being untreated or misdiagnosed, or the clinical symptoms of leptospirosis often overlap with other febrile illnesses like malaria and dengue, contributing to misdiagnosis [7]. This patient presented with several features pointing toward leptospirosis, such as occupational exposure as a farmer leading to contact with contaminated soil and water and a constellation of hepatic, renal, and hematological derangements. Weil's syndrome, which is characterized by conjunctival suffusion, jaundice, and acute kidney injury, was also evident in this patient [4].

The initial clinical profile of the patient revealed fever, abdominal discomfort, jaundice, and a marked inflammatory response (leukocytosis, elevated ESR, and CRP), which raised suspicion for acute cholangitis [8]. Cholangitis typically arises secondary to biliary obstruction (choledocholithiasis or neoplasia), as corroborated by imaging findings [9]. However, in this patient, abdominal ultrasonography showed a normal common bile duct caliber with no evidence of obstruction, rendering cholangitis substantially less likely despite the biochemical cholestatic pattern. A cholestatic liver profile in the absence of mechanical biliary obstruction warrants consideration of non‐obstructive causes of hyperbilirubinemia, including sepsis, drug‐induced liver injury, and hepatocellular injury from leptospirosis and other systemic infections [10].

The early, acute stage of infection, also known as the leptospiremic or bacteremic phase, is characterized by the sudden onset of myalgia, fever, and headache. Leptospires can enter the host bloodstream within minutes of infection. Unlike typical bacteremic agents, leptospiral lipopolysaccharide (LPS) is poorly recognized by human Toll‐like receptor 4 (TLR4), allowing the bacteria to “camouflage” themselves from the host innate immune response during the initial stages. If untreated, leptospires can translocate to distant host tissues, leading to Weil's disease, a severe form of leptospirosis [11]. Weil's syndrome specifically involves hepatic dysfunction, renal failure, and hemorrhages. Other severe forms include acute kidney injury (AKI), which can progress to chronic kidney disease (CKD), and severe pulmonary hemorrhagic syndrome (SPHS) with acute respiratory distress syndrome (ARDS). The clinical signs of severe leptospirosis, such as increased leptospiremia and multi‐organ failure, meet the criteria for a sepsis diagnosis, suggesting a dysregulated inflammatory response [12].

Renal disease in leptospirosis is dominated by acute kidney injury, usually from tubulointerstitial damage, with characteristic electrolyte losses and frequent coexistence of jaundice, thrombocytopenia, and abnormal urine findings. The laboratory pattern is tubular and systemic rather than isolated azotemia [13, 14]. Serum creatinine and urea are often markedly elevated, with pooled mean admission values of 3.6 mg/dL creatinine and 131.8 mg/dL urea in the meta‐analysis [13].

Diagnosis of leptospirosis is stage‐dependent. Although exposure history and clinical features raise suspicion, laboratory confirmation is required due to the nonspecific presentation of the disease [15]. Routine laboratory tests help in evaluating suspicion and severity, while PCR allows early confirmation before serology becomes positive. IgM serological tests serve as supportive diagnostic tools. Common findings include neutrophilia, lymphopenia, thrombocytopenia, and elevated renal and liver markers, which also correlate with disease severity [16]. Management focuses on early recognition and supportive care, with antibiotics reducing the duration of fever. In this case, early initiation of intravenous ceftriaxone led to gradual and progressive improvement in kidney function, leukocyte count, and platelet count [17, 18].

## 6 Conclusion

Leptospirosis should be considered in the differential diagnosis of cholestatic jaundice and suspected cholangitis when imaging shows no biliary obstruction, especially in patients with compatible occupational exposure. Early confirmation and prompt antibiotic therapy can lead to favorable outcomes even in the presence of multi‐organ involvement.

## Author Contributions

**Ahmed Ali Shaikh:** conceptualization, investigation, funding acquisition, writing – original draft. **Fiza Shaikh:** methodology, validation, visualization, writing – review and editing, writing – original draft. **Saad Arshad:** software, formal analysis, project administration, writing – review and editing, writing – original draft. **Tarachand Devrajani:** data curation, supervision, resources, writing – original draft, writing – review and editing. **Humayou Ahmed:** software, methodology, validation, writing – original draft. **Manal Al‐Dhaheri:** visualization, writing – review and editing, funding acquisition, writing – original draft.

## Funding

The authors have nothing to report.

## Ethics Statement

Ethical approval was not required for this case report according to institutional guidelines.

## Consent

The authors certify that an informed written consent was taken from the patient to use his data and images for the sake of research and publication. The patient was informed that his name and identity will be kept anonymous while publishing the report.

## Conflicts of Interest

The authors declare no conflicts of interest.

## Data Availability

The authors confirm that the data supporting the findings of this study are available within the article. Raw data that support the findings of this study are available from the corresponding author, upon reasonable request.
